# Meet the players: local translation at the synapse

**DOI:** 10.3389/fnmol.2014.00084

**Published:** 2014-11-11

**Authors:** Sandra M. Fernandez-Moya, Karl E. Bauer, Michael A. Kiebler

**Affiliations:** Department of Anatomy and Cell Biology, Ludwig-Maximilians-UniversityMunich, Germany

**Keywords:** RNA localization, Staufen, neurogenesis, synaptic plasticity, RNP, mRNA stability, learning and memory

## Abstract

It is widely believed that activity-dependent synaptic plasticity is the basis for learning and memory. Both processes are dependent on new protein synthesis at the synapse. Here, we describe a mechanism how dendritic mRNAs are transported and subsequently translated at activated synapses. Furthermore, we present the players involved in the regulation of local dendritic translation upon neuronal stimulation and their molecular interplay that maintain local proteome homeostasis. Any dysregulation causes several types of neurological disorders including muscular atrophies, cancers, neuropathies, neurodegenerative, and cognitive disorders.

Activity-dependent alterations in synapse structure and function are generally believed to occur during declarative memory formation (Buffington et al., 2014). This phenomenon is termed synaptic plasticity. In the hippocampus, multiple forms of synaptic plasticity, including the late-phase of long-term potentiation (L-LTP) or metabotropic glutamate receptor-dependent depression (mGluR-LTD) are dependent on dendritic protein synthesis (Kang and Schuman, 1996; Huber et al., 2002). In fact, it has been shown that components of the translational machinery, e.g., ribosomes, tRNAs, as well as translation initiation and elongation factors (Steward and Levy, 1982; Tiedge and Brosius, 1996; Sutton and Schuman, 2006), co-translational protein sorting organelles (Davis et al., 1987; Gardiol et al., 1999), the RNA-induced silencing complex (RISC) and microRNAs (miRNAs; Schratt, 2009) are all present at the synapse. These findings suggest that local translation can be regulated in a defined compartment and that this is dependent on synaptic activity. This would allow each individual synapse of a given neuron to remodel its respective synaptic proteome through alterations in protein abundance and/or protein activity (reviewed in Hanus and Schuman, 2013). Upon synaptic activation, the specific translation together with post-translational modifications, e.g., phosphorylation, methylation, or sumoylation of synaptic proteins provide a fast and an adaptive mechanism for the fine-tuning, experience-dependent formation and stabilization of newly formed synapses in neurons (Martin and Ephrussi, 2009). This is reinforced by recent studies in which genetic disruptions of signaling molecules that regulate protein translation interfere with long-term synaptic plasticity or behavioral memories (Costa-Mattioli et al., 2009). Not surprisingly, a group of developmental and adult brain disorders, including fragile X syndrome (FXS) and autism (Belmonte et al., 2004) have been linked to abnormal changes in synaptic connectivity and plasticity due to defects in protein translation in dendrites.

In this review we will first focus on recent evidence that supports a possible link between RNA transport and translational control of localized mRNAs as well as the necessity and importance of a tight translational regulation at the synapse. Furthermore, we will highlight known *trans*-acting factors responsible for mRNA localization, anchoring and translation underlying synaptic plasticity. In particular, we analyze the recent evidences that RNA-binding proteins (RBPs) and miRNAs play an important role in translational control in neurons.

## REGULATION OF mRNA TRANSLATION DURING DENDRITIC TRANSPORT

Synaptic activity has been demonstrated to trigger the active transport of mRNAs and theirs associated factors, such as RBPs or miRNAs from the cell body into (distal) dendrites (Dahm and Kiebler, 2005; Holt and Bullock, 2009; Doyle and Kiebler, 2011). But how is this transport regulated? Currently, there is little insight concerning the molecular dynamics of mRNA transport. Here, one could imagine the following working model. Upon transcription, *trans*-acting RBPs recognize and bind *cis*-acting localization elements (zipcodes), commonly located in the 3′-untranslated region (3′-UTR) of transcripts. Before its export from the nucleus to the cytoplasm, pre-mRNA is processed to a mature form and transported through the nuclear pore complex with associated factors, e.g., the exon junction complex (EJC; Le Hir et al., 2001). After its export, the RNA-protein complex undergoes maturation via the binding of additional factors, including RBPs, adaptors and/or motor proteins. This remodeling results in the packaging of the mRNA into functional transport ribonucleoprotein particles (RNPs; reviewed in Hutten et al., 2014).

Several findings indicate that the binding of specific RBPs results in translational repression of the localized transcripts (Dahm and Kiebler, 2005). For example, the zipcode binding protein 1 (ZBP1), which binds the 3′-UTR of β*-actin* mRNA, is responsible for β*-actin* mRNA localization in primary fibroblasts and neurons and prevents translation by blocking translation initiation. Only when the mRNA reaches its destination in the periphery of the cell, the release of ZBP1 from β*-actin* mRNA activates translation, through phosphorylation of ZBP1 by the non-receptor tyrosine kinase Src (Hüttelmaier et al., 2005). The translational repression of mRNAs is essential for the precise regulation of its local expression in subcellular compartments. Additional support for the model of a translational stalled mRNA comes from experiments in which protein components of the EJC, as well as other factors such as the nuclear cap-binding protein 80 (CBP80), and the nuclear poly(A)-binding protein (PABPN1), have been identified in neuronal Staufen 2 (Stau2) and Barentsz (Btz)-containing RNA granules (Fritzsche et al., 2013). These factors are routinely removed from a transcript prior or during the first round of translation (Dostie and Dreyfuss, 2002; Lejeune et al., 2002; Gehring et al., 2009). Moreover, a series of known translational repressors are enriched in Stau2 and Btz RNPs, including the proteins Fragile X mental retardation protein (FMRP), Purα, Pum2, and DDX6/Rck, as well as several components of the RISC complex (Fritzsche et al., 2013).

Once assembled, cytoplasmic RNPs are translocated along microtubules via motor proteins to synapses, a postsynaptic compartment of dendrites. However, it is not clear how mRNPs are recruited to and retained at specific, activated synapses for subsequent translation. It has been suggested that the local actin cytoskeleton and associated motor proteins anchor specific mRNAs at the synapse (Martin and Ephrussi, 2009). Alternatively, a dynein protein could serve as an anchor at the apical pole of the *Drosophila* oocyte (Delanoue and Davis, 2005). In contrast, a different scenario to anchoring can be envisioned. Specific mRNAs might be packaged into different types of mRNPs (Mikl et al., 2011; Amrute-Nayak and Bullock, 2012) depending on distinct intrinsic signals (the “RNA signature” concept), and these mRNPs then constantly patrol dendrites along microtubules in analogy to a “sushi-belt” (Doyle and Kiebler, 2011). Only if a particular synapse becomes activated, particular transcripts are released from their mRNPs, thereby becoming available for local translation at polyribosomes (reviewed in Hutten et al., 2014).

As pointed out above, synaptic activity may partially disassemble mRNPs for local translation of specific transcripts. A recent paper by Rob Singer’s laboratory has shed new light onto this point. They have shown that β*-actin* mRNAs are associated with a set of proteins and upon synaptic activation the transcripts become more accessible to FISH. These findings indicate that during its transport, a transcript is covered by different RBPs, such as FMRP or ZBP1; however, they release the transcript upon activation, thereby making it more available for translation (Buxbaum et al., 2014). In a second complementary study, the same laboratory showed that synaptic activation causes rearrangement of mRNPs, e.g., splitting into smaller particles, suggesting a partial disassembly of mRNPs prior to translation (Park et al., 2014). In this context, another interesting study suggested that neuronal mRNPs are associated with polyribosomes, which are stalled during the translation elongation phase and become re-activated upon synaptic activity (Graber et al., 2013).

In summary, all these molecular events could potentially contribute to releasing translational repression locally and allow specific mRNAs to be translated at an individual activated synapse (St Johnston, 2005; Holt and Bullock, 2009; Martin and Ephrussi, 2009; Doyle and Kiebler, 2011).

## TRANSLATION AT THE SYNAPSE

Different mechanisms on how cells control mRNA translation have been described (reviewed in Costa-Mattioli et al., 2009; Jung et al., 2014). Over the last few decades, researchers have identified a set of certain RBPs in the form of conventional translational regulators that modulate both localization and translation of functionally related groups of mRNAs, e.g., ZBP1 (Lin and Holt, 2007; Tiruchinapalli et al., 2003), FMRP (Zalfa et al., 2006; Bassell and Warren, 2008; Dictenberg et al., 2008), the cytoplasmic polyadenylation element-binding protein (CPEB; Richter, 2007) and the hnRNP A2 protein (Hoek et al., 1998). This supports the increasingly accepted hypothesis that mRNA localization and translational control are tightly linked (Hüttelmaier et al., 2005). Together, this raises a number of important questions in neuroscience: (i) how is translation switched on upon synaptic stimulation? (ii) at which stage of the mRNP assembly/localization is translation actually initiated? (iii) how are mRNAs coding for membrane components synthesized locally given the fact that there is no classical Golgi apparatus at synapses? and (iv) what kind of synaptic signals define the translation of distinct sets of specific target mRNAs?

Learning and memory require local transport of mRNAs and *de novo* synthesis of proteins. For example, LTP regulates the transport and subsequent localized synthesis of the transcript coding for the α-subunit of the calcium/calmodulin kinase II (CaMKIIα; Steward and Halpain, 1999). Simultaneously differential expression of the protein in dendrites and spines of neurons is regulated by long-term depression (LTD; Miller et al., 2002). In addition there is interesting evidence that different RBPs might play key roles during distinct forms of synaptic plasticity. For example, the Staufen 1 (Stau1) protein has been identified as a necessary regulator of transport of mRNAs in dendrites that are important for the protein synthesis-dependent late phase of LTP (late-LTP; Lebeau et al., 2011b). Surprisingly, the Stau 2 protein appears to play a different role in neurons, where it is necessary for the regulation of Microtubule associated Protein 1b (Map1b) mGluR-induced protein synthesis-dependent long-term depression (mGluR-LTD; Lebeau et al., 2011a). In this context, it is noteworthy to mention that *Arc* mRNA, another well-known dendritically localized mRNA, has been linked to both LTP and LTD (Yilmaz-Rastoder et al., 2011).

As each form of synaptic activation may exert distinct characteristics, most likely via different signaling cascades involving distinct signaling receptors, this suggest that different sets of proteins will be required for translation in order to achieve the proper form of plasticity (Vanderklish and Edelman, 2005). The serine/threonine kinase mTOR is a ubiquitous enzyme that promotes translation initiation through the phosphorylation of S6 kinase. This in turn enhances protein synthesis (Hay and Sonenberg, 2004). mTOR activity on the 4E-binding proteins (4E-BPs) permits translation initiation via disruption of 4E-BP interaction with eIF4E, a translation initiation factor that acts together with eIF4G (Pause et al., 1994; Gingras et al., 1998; Richter and Sonenberg, 2005). mTORC1 kinase, core protein of one of the two mTOR complexes, can be locally regulated in dendrites by upstream effectors activated by neuronal glutamate receptors such as NMDARs (Volk et al., 2007). mTORC1 is able to regulate a large set of transcripts in dendrites, both through Akt/PI3K and ERK signaling. For example, translation of the ion cannel *Kv1.1* mRNA is repressed by mTORC1 kinase activity (Sosanya et al., 2013). Another step of regulation is translational elongation. Here, activation of ionotropic glutamate receptors has been reported to lead to the phosphorylation of the elongation factor eEF2 by CaMKIIα thereby inhibiting its activity and repressing translation elongation (Mathews et al., 2000). These studies indicate that different forms of synaptic activity may activate distinct signaling pathways, whose final targets are (in part) RBPs that will regulate translation at individual synapses.

## TRANSLATIONAL REGULATORS

Translation can be controlled by *trans*-acting factors that associate with specific *cis*-acting elements within the 3′-UTR. Here we will highlight some recent examples of regulatory mechanisms mediated by these factors, such as RBPs, translation initiation factors and miRNAs.

### RNA-BINDING PROTEINS

There are still very few examples of RBPs that regulate specific mRNAs at synapses. It is interesting to note in this context that the majority of the best studied examples are also involved in regulating the localization of specific mRNAs from the cell body to dendrites or dendritic spines. For example, the zipcode binding protein ZBP1 promotes localization of β*-actin* from the nucleus to the synapse as well as the modulation translation by blocking 80S ribosome formation (Hüttelmaier et al., 2005). Upon phosphorylation of ZBP1 by the membrane-associated Src protein kinase, β*-actin* mRNA is released from inhibition and actively translated (Hüttelmaier et al., 2005). Another *trans*-acting factor involved in transport and translation regulation at the synapse is the CPEB (Richter, 2007). Several important dendritically localized transcripts contain cytoplasmic polyadenylation elements (CPEs). Binding of CPEB to the CPEs located in the 3′-UTRs of specific mRNAs inhibits their translation (Groisman et al., 2006). LTP induction promotes CPEB phosphorylation by the kinase Aurora A (Huang et al., 2002; Atkins et al., 2004), which in turn leads to the expulsion of the poly(A) ribonuclease PARN from the mRNP complex and the recruitment of other proteins (such as PABP). This then results in the polyadenylation of several CPE-containing mRNAs such as *CaMKII*α promoting its translation (Wu et al., 1998; Huang et al., 2002; Udagawa et al., 2012).

The translation initiation factor eIF4E recognizes the mRNA 5′-cap structure and also interacts with eIF4G, an important scaffold for the assembly of the translation initiation complex. Another mRNA-specific mechanism for regulating translation is by interfering with the binding of eIF4E, thereby blocking translational initiation. For example, the translational repressor Pumilio2 (Pum2) interacts with the 5′-cap of specific mRNAs and prevents eIF4E binding (Wharton et al., 1998; Cao et al., 2010). Additionally, eIF4G binds to PABP proteins associated to the poly(A) tail, bringing the two ends of the mRNA in close proximity via circularization, thereby facilitating translation initiation (Gray et al., 2000; Kahvejian et al., 2001; Cao et al., 2010). Interaction of eIF4G with eIF3 permits the recruitment of the 40S ribosomal subunit (reviewed in Kapp and Lorsch, 2004). It is interesting to note that in mammalian cell lines, the protein Btz has been reported to activate translation by interacting with eIF3 (Chazal et al., 2013). Together with its recently described localization in mRNA particles in dendrites (Macchi et al., 2003), this suggests a possible role for Btz in translation regulation at the synapse. Finally, the FMRP protein is an important translational regulator in neurons, affecting a large number of mRNA targets through different mechanisms, either as repressor or as activator. Upon synaptic activation, the S6 kinase-1 (S6K1) kinase has been proposed to phosphorylate FMRP resulting in translational repression of target mRNAs such as *CaMKII*α or *MAP1b* by ribosome stalling (Zalfa et al., 2003; Dolen et al., 2007; Muddashetty et al., 2007; Westmark and Malter, 2007; Gross et al., 2010; Darnell et al., 2011; Chen et al., 2014). On the other hand, dephosphorylated FMRP caused by mGluR-signaling regulates translation of *post-synaptic density 95* (*PSD-95*) in dendrites, whilst phosphorylated FMRP promotes the interaction between Ago2-miR-125a and *PSD-95* mRNA, thereby interfering with mRNA translation (Edbauer et al., 2010; Muddashetty et al., 2011).

### DENDRITIC miRNAs

A set of miRNAs has been found to be specifically enriched in dendrites and at synapses (Kosik, 2006). However, we still lack precise mechanistic insight whether these miRNAs act locally or not. Recent evidence suggests that mature miRNAs might be generated at synapses as a set of pre-miRNAs has been found there (Bicker et al., 2013). Furthermore, components of the RISC complex have been reported to associate with the endosomal compartment present at synapses (Antoniou et al., 2014). For example, upon synaptic activation interaction of the endosomal protein PICK1 with Ago2 has been described to result in the relocalization of Ago2 to endosomal compartment in dendrites. This results in reduced availability and therefore increased translation of target mRNAs of the Ago2-miRNA complex (Antoniou et al., 2014). Several distinct mRNAs are regulated by a group of specific located miRNAs at synapses. One of the most enriched miRNAs identified in dendrites, miR-26a, has been linked to microtubule assembly through inhibition of the *Map2* mRNA translation during synaptic activity (Kye et al., 2007). In primary cortical neurons, miR-124 binds several members of the Rho GTPase family thereby downregulating the SRY-box containing gene 9 (Sox9), affecting neuronal outgrowth, precursor division and differentiation (Yu et al., 2008; Cheng et al., 2009). In addition, it has been suggested that miR-125a directly affects *PSD-95* mRNA inhibiting its translation (Muddashetty et al., 2011) while miR-125b regulates NMDA receptor 2A (*GluN2A*) mRNA (Edbauer et al., 2010; Muddashetty et al., 2011; Banzhaf-Strathmann et al., 2014). Recently, overexpression of miR-125b has been linked to Tau protein hyperphosphorylation through the downregulation of the phosphatases DUSP6 and PPP1CA, implicating miR-125b in the pathogenesis of sporadic Alzheimer’s disease (Banzhaf-Strathmann et al., 2014). The brain-specific miR-134 inhibits translation of the *LimK1* mRNA, encoding a protein kinase that promotes dendritic spine development, when the synapse is not stimulated (Schratt et al., 2006; reviewed in Bicker et al., 2014).

Importantly, RBPs have been suggested to modulate miRNA-dependent silencing or degradation of target mRNAs (Filipowicz et al., 2008). FMRP phosphorylation promotes the formation of an Ago2-miR-125a complex which, as aforementioned, inhibits translation of *PSD-95* mRNA. mGluR signaling dephosphorylates FMRP, releasing the repression of miR-125a on *PSD-95* (Muddashetty et al., 2011). Another example of an RBP-miRNA-dependent translational regulation is mTORC1 signaling, HuD protein and miRNA-129. As mentioned above, mTORC1 inactivation increases the affinity of HuD for *Kv1.1* mRNA, thereby promoting its association with the *Kv1.1* transcript causing subsequent translation. On the other hand, when mTORC1 is active, HuD binds other mRNA targets such as *CaMKII*α allowing miR-129 to bind to the 3′-UTR of *Kv1.1* mRNA, inhibiting its translation (Sosanya et al., 2013). It has recently been shown that upon inactivation of mTORC1, binding of the HuD protein to *Kv1.1* overcomes miR-129 repression on *Kv1.1* mRNA. This is one of the best examples to date for the mechanism proposed by Withold Filipowicz (Filipowicz et al., 2008). This model suggests that RBPs can displace miRNAs from their mRNA target and therefore alter the stability or translation of the transcript even when the binding sites are not located in close proximity (Kundu et al., 2012; Xue et al., 2013). This mechanism has also been proposed for RBPs such as Pum 2, GW182, Ago, or HuR (Filipowicz et al., 2008; Jacobsen et al., 2010).

The precise mode of action of RBPs on miRNAs and their interaction with transcripts as well as their contribution to synaptogenesis and synaptic plasticity is not yet understood well. To date, there is some evidence of a possible competition between RBPs and miRNAs for binding to the same target mRNA (Sosanya et al., 2013). However, it is still not known whether they compete for the same binding site (e.g., by steric hindrance) or whether the binding of the RBP prevents miRNA binding to a distinct part of the transcript (e.g., by changing mRNA secondary structure). Also, RBPs themselves appear to be regulated via miRNAs. Neuronal stimulation induces miR-134 transcription through the myocyte enhancing factor 2 (Mef2) protein, which leads to the translational inhibition of Pum 2 in dendrites, promoting dendritogenesis (Khudayberdiev et al., 2009).

## OPEN QUESTIONS

It remains an open question how RBPs actually regulate the interaction of miRNAs with their biological targets inside the cell in order to regulate their translation at synapses. However, the increasing evidence that miRNAs play an important role for synaptic plasticity raises several important questions: does their malfunction cause neurological disorders? Is translation only affected if a certain miRNA is defective or absent? Are miRNAs produced directly at synapses upon demand or are they transported there in a complex with proteins as mRNPs or miRNPs? How are these miRNAs regulated once the signal is turned off? Are Argonaute proteins involved in this critical step? And finally, are RBPs and miRNAs competing for transcript binding? Which proteins are responsible for active transport and which assist in translational regulation occurring at the activated synapse?

To us, these are interesting questions that are eagerly awaiting experimental answers in the near future.

## Conflict of Interest Statement

The authors declare that the research was conducted in the absence of any commercial or financial relationships that could be construed as a potential conflict of interest.
